# Sociodemographic Predictors of Physical Functioning in the Elderly: A National Health Survey

**DOI:** 10.3390/ijerph16010037

**Published:** 2018-12-24

**Authors:** Milena Kostadinovic, Dejan Nikolic, Ivana Petronic, Dragana Cirovic, Mirko Grajic, Milena Santric Milicevic

**Affiliations:** 1 Clinical Center of Serbia, Pasterova 2, 11000 Belgrade, Serbia; mirko.grajic@med.bg.ac.rs; 2 Faculty of Medicine, University of Belgrade, 11000 Belgrade, Serbia; denikol27@gmail.com (D.N.); ivana.pm@live.com (I.P.); dragana.cirovic@udk.bg.ac.rs (D.C.); 3 Physical Medicine and Rehabilitation Department, University Children’s Hospital, 11000 Belgrade, Serbia; 4 Institute of Social Medicine, Faculty of Medicine, University of Belgrade, 11000 Belgrade, Serbia; milena.santric-milicevic@med.bg.ac.rs

**Keywords:** elderly, predictors, physical functioning

## Abstract

We aimed to evaluate the prevalence of sociodemographic factors with the presence and different degrees of walking difficulties in elderly above 65 years, and to analyze association between evaluated variables and the presence and degree of waking difficulties. In the population based study, 3540 individuals age above 65 years from Serbia were recruited. Further predictors were analyzed: gender, age, level of education, marital status, body mass index (BMI), index of well-being and place of residence. We assessed difficulty in walking half a km on level ground without the use of any aid (Group-1); and difficulty in walking up or down 12 steps (Group-2). Walking difficulties were categorized as no difficulty, some difficulty, a lot of difficulty and cannot do at all. For present difficulty significant predictors were: age (Group-1 (OR-3.022)/Group-2 (OR-3.825)), gender (Group-1 (OR-0.337)/Group-2 (OR-0.311)), educational level (Group-1 (OR-0.689)/Group-2 (OR-0.556)) and place of residence (Group-2 (OR-1.523)) while for non-performing the task, significant predictors were: age (Group-1 (OR-1.998)/Group-2 (OR-2.096)), gender (Group-1 (OR-0.629)/Group-2 (OR-0.495)), BMI (Group-1 (OR-1.219)/Group-2 (OR-1.305)), marital status (Group-1 (OR 0.764)/Group-2 (OR-0.769)), educational level (Group-1 (OR-0.679)/Group-2 (OR-0.719)) and index of well-being (Group-2 (OR-0.764)). Understanding of predictors, and their role on functional decline in elderly is of great importance for the development of specific population-based health programs to prevent further functional loss and preserve achieved functional gains.

## 1. Introduction

With the increase in life expectancy, the significance of the fourth age, that is characterized by the presence of various degrees of disability and dependence, is gaining significant importance [1,2]. The changes in functional status are reflected not only to the ones quality of life but have societal and economic consequences as well [3].

Disability, which refers to the capacity limitations in certain function performance, might be evaluated by numerous measures, with a tendency of development of additional measures and including a broader range of functional abilities [3]. Additionally, it is considered to be a dynamic variable, with inequality between functional capacity and socio-environmental changes [4]. Moreover, walking difficulty could be considered as a mobility disability measure in elderly, with the influence of both: functional capacity of the lower body and environmental domains [4]. It should be mentioned as well that evidence in the literature suggests the term preclinical stage of disability referring to the early stage in the disablement process [5], thus interventions that are aimed to identifying early declines are of great importance.

There are various aspects that are influencing the everyday lives of the aged population, including social, demographic, cultural, health and behavioral dimensions [6,7]. These multidimensional aspects refer to how complex influence they might have in functional decline in elderly. In a randomized clinical trial [8] it was stressed that exercise intervention along with early rehabilitation is effective in reversing functional decline with acute hospitalization in elderly and thus improving quality of life. Aside, physical health, it was suggested that demographic characteristics, in particular, might be associated with daily functioning [9]. Social support is another aspect that might be associated with better mental and physical health [9], thus influencing elderly activities. Further, female gender of the aged population is associated with more physical disability despite lower mortality rates [10].

Serbia among all countries in Central and Eastern Europe has estimated a maximal prevalence of inhabitants that are physically inactive or not sufficiently active [11], pointing to the not satisfactory developed individual and community culture of necessity for integration into proper physical activity.

It is know that comorbidity is frequently present in elderly [12], but so far in Serbia and countries in transition, there are a few studies dealing with physical health and needs on the representative level. Thus, mainly published studies are clinical investigations and case studies [13,14,15]. 

Therefore, in order to increase mobility in an elderly population, it is important to investigate the presence of differences of socioeconomic predictors for walking and stairs climbing, so that these predictors could be used for the environmental adaptations in order to achieve and maintain maximal functional independence. For this purpose, we split the physical activity on walking and stairs climbing (12 steps).

The aim of this study is twofold: first we aimed to evaluate the prevalence of sociodemographic factors with the presence and different degrees of walking difficulties in elderly above 65 years of life, and second, we aimed to analyze the association between evaluated variables and the presence and degree of waking difficulties in studied group.

## 2. Material and Methods

### 2.1. Participants

We evaluated 3540 elderly individuals above 65 years of age from Serbia in a population-based study. This investigation was a part of the third national study “*Istraživanje zdravlja stanovništva Srbije u 2013*” that was performed by the Ministry of Health of Republic of Serbia [16]. It investigated the health of Serbian inhabitants and was conducted in accordance with methodology and instruments of European Health Interview Survey wave 2 (EHIS wave 2) [16]. Eligible participants that were recruited into the study were informed about the study protocol and consent was obtained. The study was approved by Institutional Review Board of Faculty of Medicine, the University of Belgrade in Belgrade, Serbia (ethical code: 29/III-8). 

### 2.2. Study Criteria Selection

Census of individuals, households, and apartments in the Republic of Serbia from the 2011 year was used to form nationally representative probability sample. Populational data for Serbia (census from 2011) were used for stratification of the representative sample and 2 variables were used for initial stratums: region and settlement type. Four statistical regions as main stratums of the investigated populational sample were identified: Vojvodina; Belgrade; Sumadija and western Serbia; and southern and eastern Serbia, that were further divided into eight strata based on cities and other areas. Two-step sampling was used for percentual representation of sample distribution on the national level. First probability proportional sampling (in total 670 census areas) was done, followed by household extraction for selected census areas (10 households and three additional spare households). A simple random sample without replacement was used for household selection. There were 6500 households in total, with 3540 (24.2%) elderly above 65 years of life [16].

The inclusion criteria for study participation were private household residents of the territory of the Republic of Serbia, while exclusion criteria were collective household residents, residents of geriatric institutions, and those who refused to participate into the study. 

### 2.3. Data Processing and Preparation

Participants were divided regarding gender to males and females. Considering the age of individuals, they were divided into three groups: Group A—between 65–74 years; Group B—between 75–84 years; Group C—above 85 years [17]. There were three categories of education level, including those who finished: elementary school (≤8 years of education); high school (between 9–12 years of education); and university (>12 years of education). Regarding marital status elderly from the study were categorized into: single and married. For the evaluation of body mass index (BMI), the body height and weight measures were taken and calculated as kg/m². Following the world health organization (WHO) classification, individuals were classified into 4 BMI categories: underweight (<18.50); normal weight (18,50–24.99); overweight (≥25.00) and obese (≥30.00) [18]. Considering the place of residence, individuals were grouped into those who lived in the city or another place.

### 2.4. Wealth Index

The Demographic and Health Survey Wealth Index, or Wealth Index estimation was described in detail in previous studies, and it includes variables that are related to the property excluding income [19,20]. The wealth index of the Serbian households is ranked into five socioeconomic categories (the richest, rich, middle class, poor and the poorest) [21]. In our study, we modified this categorization into three categories: lower (poor and poorest), middle (middle class) and upper (rich and richest) [21].

### 2.5. Difficulty in Walking Assessment

We investigated two categories of walking difficulties. It is aimed to assess the individuals own capacity. The first category was: difficulty in walking half a km on level ground without the use of any aid. The question asked was: Do you have difficulty walking half a km on level ground that would be [...] without the use of any aid?; and proposed answers were: no difficulty, some difficulty, a lot of difficulty and cannot do at all/unable to do [22]. The question has to be completed with an example fitting the national context. For example: ‘the length of five football fields’ or ‘one city block’ in […] [22]. The second category was: difficulty in walking up or down 12 steps. The question asked was: Do you have difficulty walking up or down 12 steps?; and proposed answers were: no difficulty, some difficulty, a lot of difficulty and cannot do at all/unable to do [22].

## 3. Statistical Analysis

Categorical variables are presented as prevalence in percentages (%) and with 95% confidence interval (CI). For assessment of the statistical significance between these variables, we used chi squared test. For identification of factors that are independent predictors of difficulties in walking half a km on level ground without the use of any aid or difficulties in walking up or down 12 steps we used univariate logistic regression and multivariate logistic regression that included variables from univariate logistic regression with *p* < 0.05. For quantification of strength association of significant predictors and difficulties in walking half a km on level ground without the use of any aid or difficulties in walking up or down 12 steps, we used Odds ratio (OR) with 95% CI.

Four models were extracted: Model 1—Cannot perform task: walking half a km on level ground without the use of any aid; Model 2—Difficulty in walking half a km on level ground without the use of any aid with some or a lot of difficulties; Model 3—Cannot perform task: walking up or down 12 steps; Model 4—Difficulty in walking up or down 12 steps with some or a lot of difficulties.

## 4. Results

Prevalence of sociodemographic variables in relation to walking difficulties half a km on level ground without the use of any aid were presented in Table 1. Prevalence of sociodemographic variables in relation to walking difficulties up or down 12 steps, were presented in Table 2.

Significantly higher prevalence was for females versus males (some difficulty; a lot of difficulty; cannot do at all—*p* < 0.05 respectively) (Table 1 and Table 2).

There are significant differences in prevalence of age with regards to the age groups in all 4 categories of walking difficulties, with highest prevalence of elderly between 65–74 years for “no difficulty” (*p* < 0.001) (Table 1 and Table 2) and “some difficulty” (*p* < 0.01) (Table 1) and (*p* < 0.001) (Table 2) categories, and highest prevalence of elderly between 75–84 years for “a lot of difficulty” and “cannot do at all” categories (*p* < 0.05 respectively) (Table 1) and for “a lot of difficulty” (*p* < 0.001) and “cannot do at all” (*p* < 0.05) categories (Table 2). 

Married individuals were significantly more prevalent in “no difficulty” group (*p* < 0.05), while for other groups we found no significant difference in prevalence (*p* > 0.05) (Table 1 and Table 2). 

Considering educational level parameter, significant differences in prevalence of individuals with different educational levels were obtained, with highest prevalence of participants with elementary education for all 4 categories (no difficulty—*p* < 0.01; some difficulty, a lot of difficulty and cannot do at all—*p* < 0.001 respectively) (Table 1 and Table 2). 

For BMI parameter, significant differences in prevalence of different BMI categories were found, with highest prevalence of elderly that are overweight in “no difficulty” (*p* < 0.001), “some difficulty” (*p* < 0.01) and “a lot of difficulty” (*p* < 0.01) categories (Table 1 and Table 2). However, those with normal weight were most prevalent in “cannot do at all” (*p* < 0.05) category (Table 1 and Table 2).

There are significant differences in prevalence of different categories of Wealth Index in all 4 categories, with highest prevalence of lower index (no difficulty—*p* < 0.05; some difficulty, a lot of difficulty, and cannot do at all—*p* < 0.01 respectively) (Table 1) and (no difficulty; some difficulty, a lot of difficulty, and cannot do at all—*p* < 0.01 respectively) (Table 2). 

Significantly prevalent were participants from cities in “no difficulty” and “some difficulty” categories (*p* < 0.05, respectively) (Table 1) and from cities in “no difficulty” (*p* < 0.01) and “some difficulty” (*p* < 0.05) categories (Table 2).

In Table 3, sociodemographic factors significantly associated with the presence and degree of difficulty in walking half a km on level ground without the use of any aid by univariate and multivariate analysis were presented. After applying variables that were significantly associated with evaluated Models from univariate logistic regression in multivariate logistic regression, age was significantly associated with Model 1 (OR = 3.022) and Model 2 (OR = 1.998), gender was significantly associated with Model 1 (OR = 0.337) and Model 2 (OR = 0.629), BMI was significantly associated with Model 2 (OR = 1.219), marital status was significantly associated with Model 2 (OR = 0.764) and educational level was significantly associated with Model 1 (OR = 0.689) and Model 2 (OR = 0.679).

In Table 4, sociodemographic factors significantly associated with the presence and degree of difficulty in walking up or down 12 steps by univariate and multivariate analysis were presented. After applying variables that were significantly associated with evaluated Models from univariate logistic regression in multivariate logistic regression, age was significantly associated with Model 3 (OR = 3.825) and Model 4 (OR = 2.096), gender was significantly associated with Model 3 (OR = 0.311) and Model 4 (OR = 0.495), BMI was significantly associated with Model 4 (OR = 1.305), marital status was significantly associated with Model 4 (OR = 0.769), educational level was significantly associated with Model 3 (OR = 0.556) and Model 4 (OR = 0.719), wealth index was significantly associated with Model 4 (OR = 0.764) and place of residence was significantly associated with Model 3 (OR = 1.523).

**Figure 1:**
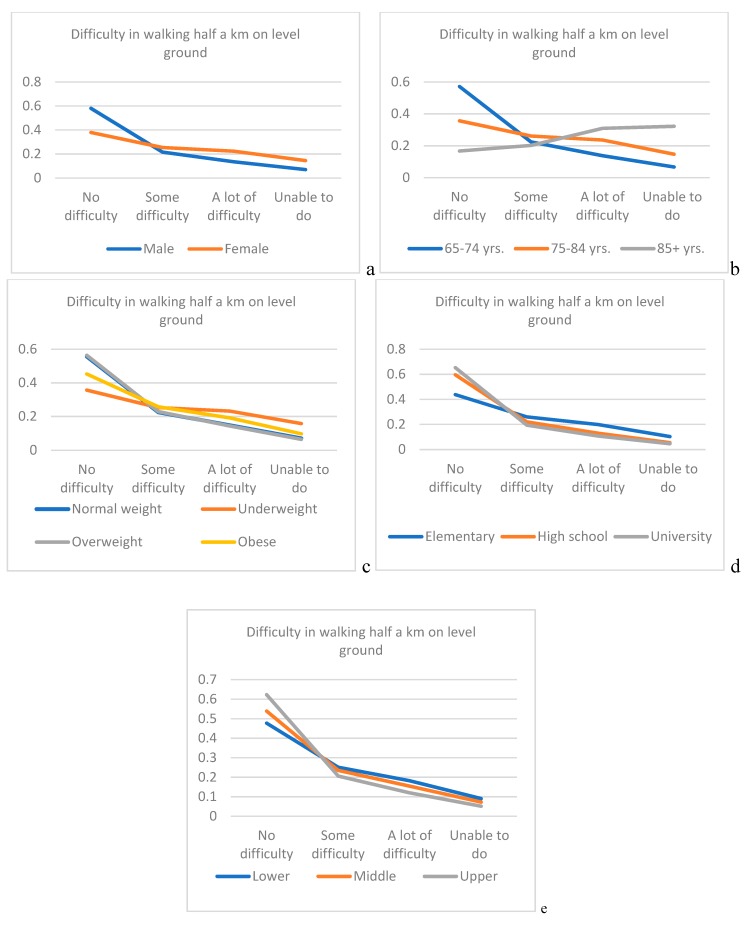
Predicted probability for tested variables associated with the presence and degree of difficulty in walking half a km on level ground without the use of any aid. (**a**) gender; (**b**) age; (**c**) body mass index (BMI); (**d**) educational level; (**e**) Wealth Index.

In Figure 1, we presented predicted probability for tested variables associated with the presence and degree of difficulty in walking half a km on level ground without the use of any aid.

**Figure 2:**
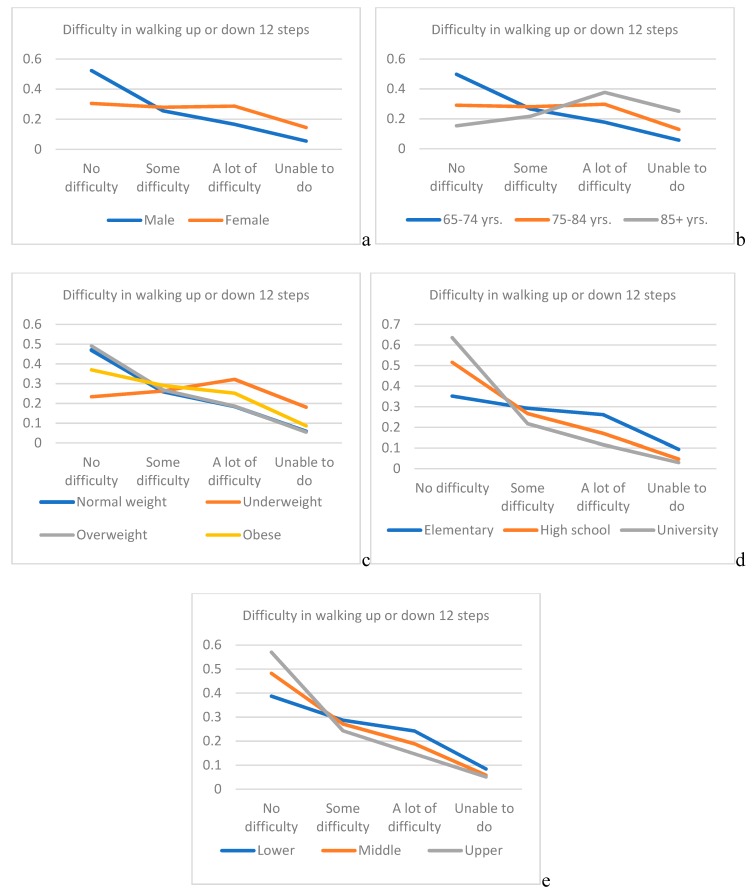
Predicted probability for tested variables associated with the presence and degree of difficulty in walking up or down 12 steps. (**a**) gender; (**b**) age; (**c**) BMI; (**d**) educational level; (**e**) Wealth Index).

In Figure 2, associated with the presence and degree of difficulty in walking up or down 12 steps. Predicted probability for elderly above 85 years of life for the category—not to have difficulties in walking half a km on level ground without the use of any aid or, up or down 12 steps is almost 3.5 times lesser than for elderly between 65–74 years (0.17 vs. 0.57 (Figure 1); 0.15 vs. 0.50 (Figure 2)).

## 5. Discussion

For a better understanding of predictors and risk factors, as well as their role on functional decline and physical activity behavior in elderly is of great importance for the development of specific population-based health programs in order to prevent further functional loss and preserve achieved functional gains. These programs will have great benefits of better physical fitness promotion, more independent lifestyle and ultimately overall quality of life in elderly.

We have demonstrated that age is the most significant independent predictor of walking difficulty among elderly, with the most prevalent age between 65–74 for the “some difficulty” and most prevalent age between 75–84 years for “a lot of difficulty” and for elderly who cannot perform the task. In our study, no difficulties in performing walking half a km on level ground without the use of any aid was most prevalent in individuals that were between 65–74 years, with just above 30%, while for the task walking up or down 12 steps, no difficulties were as well in the same age group most prevalent with just below 30%. In elderly between 75–84 years of age, prevalence of “no difficulty” category in performing both types of walking difficulties tasks was reduced to more than double. In the study of Shumway-Cook et al., it was noticed that older age was associated with an increase in severity of mobility limitation [23]. Considering the two types of evaluated walking difficulties, we have shown that there are for same age groups different range in prevalence with regards to the degree of walking difficulty. Moreover, in our study, it was shown that the age is a stronger independent predictor for difficulty in walking up or down 12 steps, than walking half a km on level ground without the use of any aid. Such observation could be justified by the fact that walking on steps is much demanding particularly in domains of strength and balance. We have demonstrated that in elderly age between 65 and 84 years, there is an inverse effect of predicted probability between age and the increase in the degree of difficulty in walking half a km on level ground, while the opposite trend was noticed for those above 85 years. Considering the evaluation of the degree of difficulty in walking up or down 12 steps, the inverse effect remained only for those age between 65–74 years, while for those between 75–84 years, and for those 85 and above such effect was noticed between two most severe degrees.

Further, we have shown that the second strongest significantly independent predictor of walking disability in the aged population is gender, particularly females. Considering the task of walking difficulties half a km on level ground without the use of any aid, “no difficulty” category was most prevalent in both genders, but for the task that demands higher intensity: walking difficulties up or down 12 steps, the category “no difficulty” remained most prevalent in males but, for females in such case “some difficulty” category was most prevalent. It was stressed previously that females are at greater risk of impaired physical functioning, due to the numerous factors, among them: lower muscle strength, mass and power as well as increased adiposity compared to males [24,25]. In the study of Straight et al., it was pointed that strongest predictors for physical functioning of lower extremities particularly in elderly females are muscle quality and relative adiposity [26]. In line with such findings, it was shown in our study that the gender is a stronger independent predictor for difficulty in walking up or down 12 steps, than walking half a km on level ground without the use of any aid. Female gender was just below three times at higher risk than males for having some kind of difficulty in walking on level ground and slightly above three times greater risk than males for having some kind of difficulty in walking on steps. Same trend but twice in favor of females was noticed for those who cannot perform at all both tasks. There is an inverse effect of predicted probability for both genders and the increased of the degree of difficulty in walking half a km on level ground with higher effects for male. Considering the degree of difficulty in walking up or down 12 steps, for male inverse effect remained, while for females such effect was noticed between two most severe degrees.

In our study, BMI was noticed as significant independent predictor for both walking difficulties on ground level and on steps, but only for elderly who cannot perform these tasks at all, where it was stronger independent predictor for difficulty in walking up or down 12 steps than walking half a km on level ground without the use of any aid for those. There are conflicting reports considering the BMI and its association with daily life functioning. While some researchers point that the better daily life functioning is associated with higher BMI [27], others stress that those who are underweight or obese has more limitations [28,29]. Our findings point that overweight elderly was most prevalent in all evaluated categories for both tasks of walking difficulties except for the category “cannot do at all” where most prevalent were those with normal weight. Regarding predicted variability for BMI there is inverse effect with the degree of difficulty in walking half a km on level ground for all tested BMI groups, while inverse effect remained for all BMI groups except for underweight where such effect was noticed between two most severe degrees.

Considering marital status, our findings stress that it is a significant independent predictor for both tasks of walking difficulties but only for the category “cannot do at all”, with similar OR between tasks. In the category “no difficulty” in performing both tasks of walking difficulties, married elderly were most prevalent, while in categories with present difficulties we found non-significant differences in prevalence considering marital status. In the study of Kaplan et al., it was shown that marital status was significantly associated with the change in physical functioning in elderly [30]. Moreover, in the study of Cakar et al., it was pointed out that marriage was associated with better physical functioning in elderly [31].

In the study of Chad et al., it was noticed that higher levels of education were associated with better physical activity in elderly [32]. Such findings might be justified by the fact that the individuals with higher educational levels could have numerous advantages which in fact promotes the physical activity in terms of increased knowledge, stronger sense of personal control and better access to various resources [33]. Our findings confirm that educational level is a significant independent predictor of walking difficulty in elderly both for walking half a km on level ground without the use of any aid, and for walking up or down 12 steps, with most prevalent elementary education in all categories. There is an inverse effect of predicted probability between levels of education and both degrees of difficulty in walking (for half a km on level ground and for up or down 12 steps).

Wealth Index in our study was shown to be significant independent predictor only for the category “cannot do at all” for difficulty in walking up or down 12 steps task, while place of residence was significant independent predictor only for categories with present difficulties for the task: difficulty in walking up or down 12 steps. We have demonstrated an inverse effect of predicted probability between wealth index and both degrees of difficulty in walking (for half a km on level ground and for up or down 12 steps).

In conclusion, this study on a representative sample of elderly in Serbia demonstrated that female gender is more prevalent in elderly with some disability. Younger elderly is more prevalent in group with some disability, while single individuals and those from non-urban areas were more prevalent in groups with more severe disabilities. Lower education level and lower wealth index were significantly prevalent in all of the evaluated categories. Considering evaluated sociodemographic factors, development of adequate social support for older elderly, females, those living alone, as well as those with lower educational levels and lower wealth index is necessary to improve mobility, functionality and over quality of life. Furthermore, social support should be on a national level in order to increase mobility awareness.

## Figures and Tables

**Table 1:** Prevalence of sociodemographic variables in relation to walking difficulties half a km on level ground without the use of any aid.

Variables	Difficulty in Walking Half a km on Level Ground without the Use of any Aid Prevalence (%) and 95 CI				*p*
	No Difficulty	Some Difficulty	A lot of Difficulty	Cannot do at all	
Gender					
Female	21.1 (19.8–22.4)	15.4 (14.2–16.6)	12.1 (11.0–13.2)	8.2 (7.3–9.1)	<0.01
Male	25.3 (23.9–26.7)	8.4 (7.4–9.3)	6.5 (5.7–7.3)	3.0 (2.4–3.6)	<0.01
*p*	>0.05	<0.05	<0.05	<0.05	
Age					
65–74 years	31.6 (30.1–33.1)	12.2 (11.1–13.3)	7.9 (7.0–8.8)	3.5 (2.9–4.1)	<0.001
75–84 years	13.8 (12.7–15.0)	10.5 (9.4–11.5)	9.0 (8.1–10.0)	5.8 (5.0–6.6)	<0.05
≥85 years	1.0 (0.7–1.3)	1.2 (0.8–1.5)	1.6 (1.2–2.0)	1.9 (1.4–2.3)	>0.05
*p*	<0.001	<0.01	<0.05	<0.05	
Marital status					
Single	16.1 (14.9–17.3)	11.8 (10.8–12.9)	10.2 (9.2–11.2)	6.3 (5.5–7.1)	<0.05
Married	30.3 (28.8–31.8)	11.9 (10.9–13.0)	8.4 (7.5–9.3)	4.9 (4.2–5.6)	<0.05
*p*	<0.05	>0.05	>0.05	>0.05	
Education level					
Elementary	19.5 (18.2–20.8)	15.2 (14.0–16.4)	12.5 (11.4–13.5)	8.1 (7.2–9.0)	<0.01
High school	18.1 (16.9–19.4)	6.1 (5.3–6.9)	4.7 (4.0–5.4)	2.3 (1.8–2.8)	<0.001
University	8.8 (7.9–9.7)	2.5 (2.0–3.0)	1.5 (1.1–1.9)	0.8 (0.5–1.1)	<0.01
*p*	<0.01	<0.001	<0.001	<0.001	
body mass index (BMI)					
Underweight	0.5 (0.3–0.8)	0.5 (0.3–0.7)	0.3 (0.1–0.5)	0.3 (0.1–0.5)	<0.05
Normal weight	14.0 (12.8–15.1)	5.3 (4.6–6.0)	3.5 (2.9–4.1)	2.0 (1.5–2.4)	<0.01
Overweight	16.6 (15.3–17.8)	7.1 (6.3–8.0)	4.5 (3.8–5.1)	1.6 (1.2–2.0)	<0.001
Obese	6.9 (6.1–7.7)	3.8 (3.1–4.4)	3.1 (2.5–3.6)	1.5 (1.1–1.9)	<0.01
*p*	<0.001	<0.01	<0.01	<0.05	
Wealth Index					
Lower	22.1 (20.7–23.5)	14.5 (13.4–15.7)	12.1 (11.0–13.2)	7.1 (6.2–7.9)	<0.05
Middle	8.9 (8.0–9.8)	3.9 (3.3–4.5)	3.1 (2.5–3.7)	1.9 (1.5–2.4)	<0.05
Upper	15.4 (14.2–16.6)	5.3 (4.6–6.1)	3.4 (2.8–4.0)	2.2 (1.7–2.7)	<0.01
*p*	<0.05	<0.01	<0.01	<0.01	
Place of residence					
City	27.5 (26.0–29.0)	13.0 (11.9–14.1)	8.4 (7.5–9.3)	4.9 (4.2–5.6)	<0.001
Another place	18.9 (17.6–20.2)	10.8 (9.8–11.8)	10.2 (9.2–11.2)	6.3 (5.5–7.1)	<0.01
*p*	<0.05	<0.05	>0.05	>0.05	

**Table 2:** Prevalence of sociodemographic variables in relation to walking up or down 12 steps.

Variables	Difficulty in Walking up or down 12 Steps Prevalence %, (95 CI)				*p*
	No Difficulty	Some Difficulty	A lot of Difficulty	Cannot do at all	
Gender					
Female	16.6 (15.4–17.9)	17.2 (16.0–18.4)	16.0 (14.8–17.2)	7.0 (6.2–7.9)	<0.01
Male	23.1 (21.7–24.4)	9.8 (8.8–10.8)	7.7 (6.8–8.6)	2.3 (1.8–2.8)	<0.01
*p*	<0.05	<0.05	<0.05	<0.05	
Age					
65–74 years	27.5 (26.1–29.0)	14.4 (13.3–15.6)	10.2 (9.2–11.2)	3.0 (2.5–3.6)	<0.001
75–84 years	11.4 (10.3–12.4)	11.1 (10.1–12.1)	11.5 (10.5–12.6)	5.1 (4.4–5.9)	<0.05
≥85 years	0.8 (0.5–1.1)	1.4 (1.0–1.8)	1.9 (1.5–2.4)	1.5 (1.1–1.9)	>0.05
*p*	<0.001	<0.001	<0.001	<0.05	
Marital status					
Single	13.0 (11.9–14.1)	13.0 (11.9–14.1)	13.0 (11.9–14.1)	5.5 (4.7–6.2)	<0.05
Married	26.7 (25.2–28.1)	14.0 (12.8–15.1)	10.7 (9.7–11.7)	4.2 (3.5–4.8)	<0.01
*p*	<0.05	>0.05	>0.05	>0.05	
Education level					
Elementary	16.0 (14.8–17.2)	15.8 (14.6–17.0)	16.4 (15.1–17.6)	7.1 (6.32–8.0)	<0.01
High school	15.1 (14.0–16.3)	8.4 (7.4–9.3)	5.7 (5.0–6.5)	1.9 (1.5–2.4)	<0.001
University	8.6 (7.6–9.5)	2.8 (2.3–3.40)	1.6 (1.2–2.0)	0.6 (0.3–0.8)	<0.001
*p*	<0.01	<0.001	<0.001	<0.001	
BMI					
Underweight	0.4 (0.2–0.6)	0.5 (0.3–0.8)	0,3 (0,1–0,5)	0.4 (0.2–0.6)	>0.05
Normal weight	12.6 (11.5–13.7)	5.9 (5.2–6.7)	4.4 (3.8–5.1)	1.8 (1.3–2.2)	<0.001
Overweight	14.2 (13.1–15.4)	8.6 (7.7–9.5)	5.5 (4.8–6.3)	1.3 (1.0–1.7)	<0.001
Obese	5.7 (4.9–6.4)	4.1 (3.4–4.7)	4.3 (3.6–5.0)	1.2 (0.8–1.5)	<0.05
*p*	<0.001	<0.001	<0.01	<0.05	
Wealth Index					
Lower	18.3 (17.0–19.5)	15.4 (14.2–16.6)	15.9 (14.7–17.1)	6.2 (5.4–7.0)	<0.01
Middle	7.7 (6.80–8.5)	4.8 (4.1–5.5)	3.6 (3.0–4.2)	1.8 (1.3–2.2)	<0.01
Upper	13.8 (12.6–14.9)	6.8 (5.9–7.6)	4.2 (3.5–4.8)	1.7 (1.3–2.1)	<0.001
*p*	<0.01	<0.01	<0.01	<0.01	
Place of residence					
City	23.4 (22.0–24.8)	15.4 (14.2–16.6)	10.8 (9.7–11.8)	4.2 (3.5–4.8)	<0,001
Another place	16.3 (15.1–17.5)	11.6 (10.5–12.6)	12.9 (11.8–14.0)	5.5 (4.7–6.2)	<0.05
*p*	<0.01	<0.05	>0.05	>0.05	

**Table 3:** Sociodemographic factors significantly associated with the difficulty in walking half a km on level ground without the use of any aid.

Sociodemographic Variables	Model 1 OR (95% CI)	Model 2 OR (95% CI)
Univariate logistic regression		
Age	3.934 * (3.258–4.750)	2.172 * (1.912–2.469)
Gender	0.303 * (0.238–0.387)	0.450 * (0.390–0.520)
BMI	1.034 (0.849–1.260)	1.154 ** (1.038–1.284)
Marital status	0.412 * (0.330–0.515)	0.492 * (0.426–0.568)
Education level	0.390 * (0.324–0.470)	0.517 * (0.466–0.573)
Wealth Index	0.673 * (0.589–0.770)	0.683 * (0.628–0.742)
Place of residence	1.887 * (1.512–2.355)	1.433 * (1.244–1.650)
Multivariate logistic regression (step forward)		
Age	3.022 * (2.328–3.923)	1.998 * (1.699–2.348)
Gender	0.337 * (0.239–0.474)	0.629 * (0.521–0.759)
BMI	-	1.219 *** (1.088–1.364)
Marital status	-	0.764 *** (0.633–0.923)
Education level	0.689 *** (0.549–0.865)	0.679 *** (0.597–0.773)
Wealth Index	-	-
Place of residence	-	-

* *p* < 0.001; ** *p* < 0.05; *** *p* < 0.01.

**Table 4:** Sociodemographic factors significantly associated with the difficulty in walking up or down 12 steps.

Sociodemographic Variables	Model 3 OR (95% CI)	Model 4 OR (95% CI)
Univariate logistic regression		
Age	4.101 * (3.335–5.042)	2.211 * (1.941–2.518)
Gender	0.269 * (0.207–0.349)	0.381 * (0.330–0.439)
BMI	0.889 (0.718–1.101)	1.229 * (1.107–1.365)
Marital status	0.373 * (0.293–0.475)	0.463 * (0.401–0.536)
Education level	0.347 * (0.282–0.425)	0.495 * (0.447–0.547)
Wealth Index	0.610 * (0.526–0.708)	0.679 * (0.625–0.736)
Place of residence	1.889 * (1.488–2.398)	1.348 * (1.170–1.552)
Multivariate logistic regression (step forward)		
Age	3.825 * (3.063–4.775)	2.096 * (1.779–2.470)
Gender	0.311 * (0.232–0.416	0.495 * (0.410–0.596)
BMI	-	1.305 * (1.165–1.463)
Marital status	-	0.769 ** (0.636–0.929)
Education level	0.556 * (0.444–0.696)	0.719 * (0.631–0.819)
Wealth Index	-	0.764 * (0.685–0.852)
Place of residence	1.523 ** (1.141–2.033)	-

* *p* < 0.001; ** *p* < 0.01.
